# Intermediate monocytes in blood correlate with subclinical vascular changes in lupus nephritis

**DOI:** 10.1136/lupus-2024-001432

**Published:** 2025-02-06

**Authors:** Shivani Garg, Sara S McCoy, Izzy Hartel, Abigail Muhlstock, Amish N Raval, Christie Bartels

**Affiliations:** 1 Medicine, Rheumatology, University of Wisconsin-Madison School of Medicine and Public Health, Madison, Wisconsin, USA; 2 Department of Medicine, Division of Cardiology, University of Wisconsin-Madison School of Medicine and Public Health, Madison, Wisconsin, USA

**Keywords:** Cardiovascular Diseases, Lupus Nephritis, Risk Factors

 Atherosclerosis cardiovascular disease (ASCVD)-related mortality is higher in patients with lupus nephritis (LN) compared with those without nephritis (11.7 vs 3.6 per 100 patient-years).^1^ Accelerated atherosclerosis risk in LN cannot be fully explained by traditional risk factors. Many hypothesise that dysregulated cytokine profiles and altered cellular subsets may trigger endothelial dysfunction and plaque destabilisation, accelerating atherosclerosis risk in LN.^2^ We recently established that moderate-severe renal arteriosclerosis (ASCL) in kidney biopsies is a marker of premature endothelial dysfunction in LN and is associated with three times higher 10-year clinical ASCVD occurrence, underscoring its role as potential proxy for clinical ASCVD.^3^ Yet, long-term monitoring of renal ASCL is difficult, as it requires baseline and repeat invasive kidney biopsies. Therefore, a cellular surrogate biomarker of renal vascular changes that may predict clinical ASCVD risk is needed in LN.

The axiom in ASCVD science supports circulating monocytes as a cellular hallmark of atherosclerosis.^2^ A major subset (80–90%) of circulating monocytes are classical (CD14+CD16-) monocytes. While non-classical (CD14variable CD16++) and intermediate (CD14+CD16+) monocytes, constituting 10–20% of circulating monocytes, are attuned towards vascular patrolling and are pro-atherogenic. These pro-atherogenic (CD16+CD14variable) monocyte subpopulations are two times higher in patients with lupus.^2^ Previous work highlighted a correlation between pro-atherogenic (CD16+CD14variable) monocytes and endothelial dysfunction and plaque destabilisation in lupus and patients with stable coronary artery disease.^2 4^ Similar associations remain underexplored in patients with LN, who have the greatest need for early identification of vascular disease. Additionally, associations between renal vascular (renal ASCL) changes and monocyte subpopulations need to be examined in LN.

To examine the associations between circulating monocyte subpopulations and renal ASCL, we collected blood samples from 26 patients undergoing a kidney biopsy to confirm LN diagnosis or flare. We used standard gating technique for flow cytometry to quantify monocyte subpopulations in blood: classical (CD16- CD14+), non-classical (CD16++CD14variable) and intermediate (CD16+CD14+). We re-read all kidney biopsy slides using Banff criteria to grade renal ASCL as percent luminal area narrowing of renal arteries, and abstracted LN chronicity and activity indices from LN reports. We abstracted sociodemographics and PREVENT ASCVD risk factors (age, sex, zip code, kidney function, lipids, blood pressure, HgbA1c, urine protein creatinine ratio, lipid-lowering therapies, antihypertensives)^5^ and LN medications (hydroxychloroquine, prednisone, immunosuppressants). A 10-year PREVENT ASCVD risk score was calculated for each patient. Spearman’s rank correlation analysis was performed to test correlations between the proportion of monocyte subpopulations and continuous renal ASCL in all patients and two subgroups (incident LN, LN flare). Next, using linear regression modelling, we tested the associations between monocyte subpopulation proportions and continuous renal ASCL, controlling for other variables. Finally, to estimate area-under-the-curve (AUC) for predicting moderate-severe renal ASCL (>25% luminal narrowing), we compared logistic regression models including 10-year PREVENT ASCVD risk score alone and with monocyte subpopulations. A sensitivity analysis using a composite outcome variable, including an ASCVD event (eg, angina, stroke, coronary artery disease) or moderate-severe renal arteriosclerosis (>25%), was performed to re-estimate AUC.

Among 26 patients recruited over 18 months, 9 had incident LN and 17 had a renal flare. Mean age was 38±15, 81% were female, 62% were of White race and 27% were of Asian race (online supplemental table 1). Mean (SD) counts (cells/μL) for classical, intermediate and non-classical monocytes were 340 (430), 104 (110) and 24 (36), respectively. Mean renal ASCL was 16±17%; 27% had moderate-severe renal ASCL (>25%).

In the full cohort (n=26), a positive correlation was noted between the proportion of intermediate (CD16+CD14+) monocytes and continuous renal ASCL (Cor=0.56, 95% CIs=0.22 to 0.78, p value 0.003, figure 1A). While a moderately negative correlation was noted between classical (CD16- CD14+) monocytes and renal ASCL (Cor=−0.41, 95% CIs −0.04 to –0.69, p value 0.04, figure 1A), and no correlation was noted between non-classical monocytes and renal ASCL (Cor=0.28, p value 0.161, figure 1A). Additionally, mean (SD) intermediate monocyte count was higher by 130 (91) in patients with renal ASCL >25% vs ≤25% (figure 1B).

**Figure 1:**
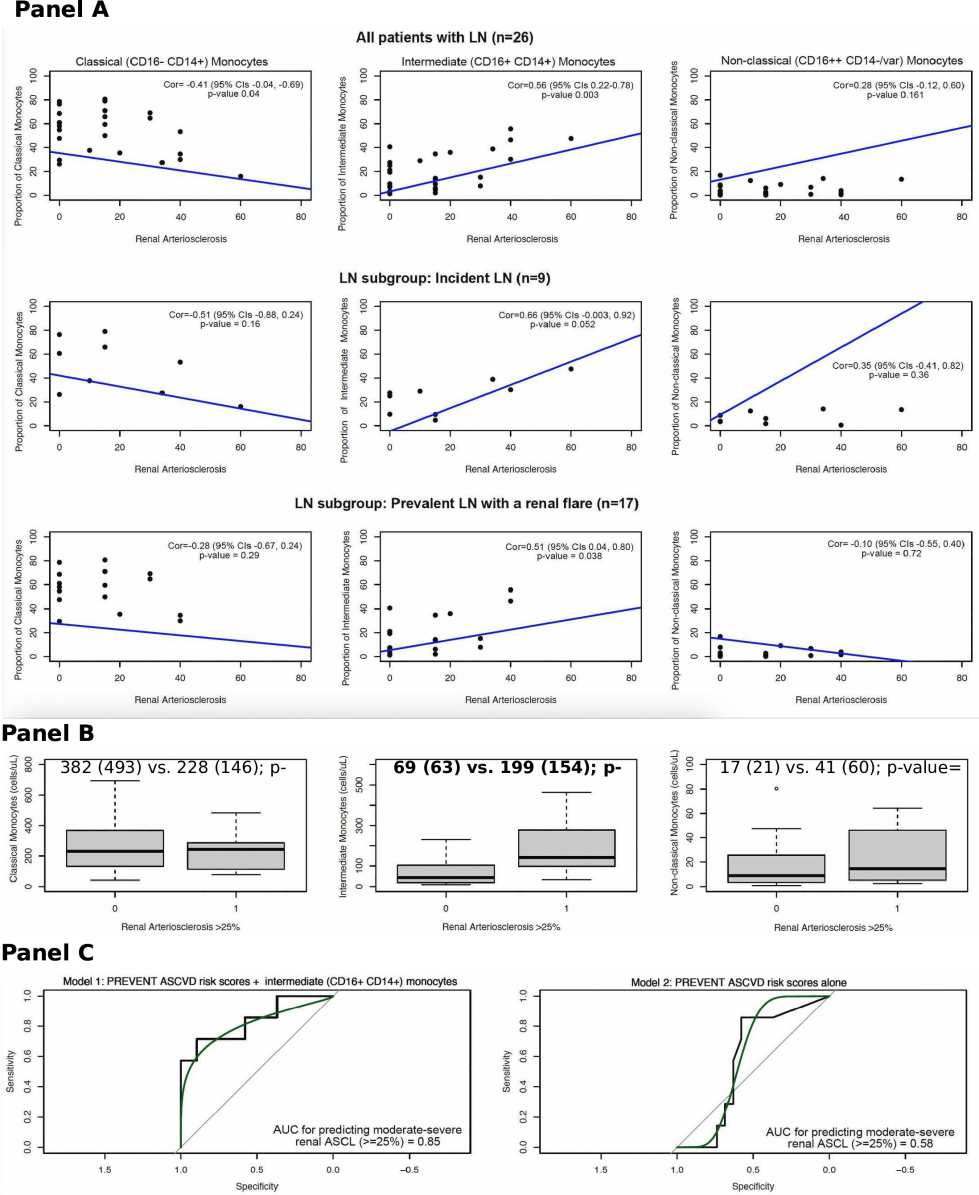
Panel A: Correlation between monocyte subpopulations and continuous renal arteriosclerosis (renal ASCL) in all patients, incident lupus nephritis (LN) and LN flares. Panel B: Boxplot showing differences in mean (SD) monocyte subpopulation counts in patients without versus with moderate-severe renal arteriosclerosis >25% (n=26, all patients). Panel C: Area-under-the-curve (AUC) for predicting moderate-severe renal ASCL (≥25%) using two models: (1) including PREVENT ASCVD risk scores and the proportion of intermediate monocyte subpopulations; (2) PREVENT ASCVD risk scores alone.

In the full cohort, a strong linear positive association was noted between continuous renal ASCL and intermediate monocyte proportions: 1% increase in the intermediate monocyte proportion was associated with 0.61% increase in renal ASCL (online supplemental table 2). Given the small sample size, we performed sequential linear regression modelling, even in reduced models with limited covariables, 1% increase in the intermediate monocyte proportion was associated with 0.58–0.61% increase in renal ASCL (online supplemental table 3). Using logistic regression, we noted that every 1% increase in the intermediate monocyte proportion was associated with 1.1-fold higher odds of renal ASCL >25% after adjusting for PREVENT ASCVD scores (online supplemental file 2). After adding the proportion of intermediate monocytes to PREVENT ASCVD scores, the AUC for predicting renal ASCL >25% increased from 0.58 (95% CIs 0.35 to 0.81) to 0.85 (95% CIs 0.63 to 1.0) (figure 1C). Cross-validating using a 50–50 spilt revealed an even higher AUC of 0.93 (95% CIs 0.76 to 1.0) of the model including intermediate monocyte proportions. Lastly, in our sensitivity analysis including ASCVD events or moderate-severe ASCL, AUC improved from 56% to 74% after including intermediate monocytes with PREVENT scores (online supplemental table 4).

Given the exploratory nature of the study and limited sample size, we do acknowledge limitations. First, we cannot draw strong conclusions and establish causal links. Thus, our findings need to be validated in larger LN cohorts. Second, this cross-sectional study did not examine change in circulating intermediate monocytes over time with therapy, which will be assessed in a future study. Finally, only two ASCVD events occurred in our cohort; future studies will examine associations between intermediate monocytes and ASCVD events over time.

There is evidence supporting a potential role of altered immune cells in the pathogenesis of atherosclerosis in lupus, and now in LN. Our study delivers early evidence on the potential correlation between circulating intermediate (CD16+CD14+) monocytes and renal vascular changes underscoring a possible role in driving vasculopathy in LN. Future longitudinal studies need to further assess the role of circulating intermediate (CD16+CD14+) monocytes in ASCVD risk assessment and management in LN. Circulating monocytes can be measured in a peripheral blood draw but could add to medical costs. Therefore, future studies should estimate intermediate monocyte cut-offs that associated with higher ASCVD risk and subgroups who would benefit most. These data will inform net reclassification estimate to guide cost-benefit analysis of using circulating monocytes in all patients with LN versus high-risk subgroups.

## supplementary material

10.1136/lupus-2024-001432 online supplemental file 1

10.1136/lupus-2024-001432 online supplemental file 2

10.1136/lupus-2024-001432 online supplemental file 3

10.1136/lupus-2024-001432 online supplemental file 4

## Data Availability

All data relevant to the study are included in the article or uploaded as supplementary information.
