# Supplementary material for: Intermediate monocytes in blood correlate with subclinical vascular changes in lupus nephritis
Source: Lupus Sci Med. 2025 Feb 6;12(1):e001432. doi: 10.1136/lupus-2024-001432 (PMC11804201; doi:10.1136/lupus-2024-001432)
Supplement: online supplemental file 1 [file lupus-12-1-s001.docx]

| Supplementary File 1. Patient Characteristics at LN Diagnosis or LN Flare | |
| --- | --- |
| Key Variables at LN Diagnosis or LN Flare |  |
| Age in years, mean (SD) | 37.7 (15.4) |
| Female, n(%) | 21 (81%) |
| Race or Ethnic groups |  |
| White, n(%) | 16 (62%) |
| Asian, n(%) | 7 (27%) |
| Black, n(%) | 3 (12%) |
| Traditional ASCVD Risk Factors |  |
| History of Hypertension, n(%) | 16 (62%) |
| History of Diabetes, n(%) | 3 (12%) |
| Active smoking, n(%) | 2 (8%) |
| Body Mass Index in kg/m^2^, mean (SD) | 31 ± 8 |
| Average Systolic Blood Pressure in mm/Hg, mean (SD) | 129 ± 13 |
| HgbA1c in mg/dL, mean (SD) | 5.7 ± 1.1 |
| Low Density Lipoprotein in mg/dL, mean (SD) | 99 ± 39 |
| Triglycerides in mg/dL, mean (SD) | 130 ± 46 |
| Urine Protein Creatinine Ratio in mg/mg, mean (SD) | 2.2 ± 2.6 |
| Estimated Glomerular Filtration Rate in ml/min/1.73m^2^, mean (SD) | 69 ± 28 |
| PREVENT 10-year ASCVD risk score*, mean (SD) | 3.1 (5.2) |
| Hydroxychloroquine Dose in mg, mean (SD) | 312 (79) |
| Prednisone Dose in mg, mean (SD) | 12 (16) |
| Immunosuppressants |  |
| None, n(%) | 3 (12%) |
| At least 1, n(%) | 12 (46%) |
| At least 2, n(%) | 3 (12%) |
| At least 3, n(%) | 4 (15%) |
| At least 4, n(%) | 4 (15%) |
| LN Chronicity Index, mean (SD) | 2.5 (2.8) |
| LN Activity Index, mean (SD) | 3.7 (4.8) |
| Incident LN, n(%) | 9 (35%) |
| LN flare, n(%) | 17 (65%) |
| Circulating Monocyte Subpopulations in cells/uL |  |
| Classical CD14++ CD16- Monocyte count in cells/uL, mean (SD) | 340 (430) |
| Intermediate CD14+ CD16+ Monocyte count in cells/uL, mean (SD) | 104 (110) |
| Non-Classical CD14var CD16++ Monocyte count in cells/uL, mean (SD) | 24 (36) |

**PREVENT ASCVD risk score calculated using AHA endorsed PREVENT risk calculator which uses information on age, sex, zip-code, body mass index, kidney function, smoking, systolic blood pressure, lipid profile, hgba1c, urine protein creatinine ratio, use of anti-hypertensive medications, active lipid lowering therapies to calculate a 10-year clinical ASCVD risk.*
