# Supplementary material for: Intermediate monocytes in blood correlate with subclinical vascular changes in lupus nephritis
Source: Lupus Sci Med. 2025 Feb 6;12(1):e001432. doi: 10.1136/lupus-2024-001432 (PMC11804201; doi:10.1136/lupus-2024-001432)
Supplement: online supplemental file 2 [file lupus-12-1-s002.docx]

| Table 1A. Multivariable linear regression modelling showing factors associated with continuous subclinical renal arteriosclerosis (renal ASCL) in patients with LN | | | | | | |
| --- | --- | --- | --- | --- | --- | --- |
| **All Patients (n=26)** | | | **New LN (n=9)** | | **LN Flares (n=17)** | |
| **Variables** | **Estimate (95% CIs)** | **p** | **Estimate (95% CIs)** | **p** | **Estimate (95% CIs)** | **p** |
| PREVENT 10-year ASCVD risk estimate | -0.23 (-1.43, 0.96) | 0.69 | -0.56 (-2.26, 1.51) | 0.44 | -0.56 (-2.01, 1.58) | 0.80 |
| NIH LN activity score | -0.61 (-1.89, 0.67) | 0.33 | - | - | - | - |
| NIH LN chronicity score | -1.41 (-3.73, 0.92) | 0.22 | - | - | - | - |
| Prednisone dose at diagnosis, 1 mg increase | -0.06 (-0.42, 0.31) | 0.75 | - | - | - | - |
| HCQ dose at diagnosis, 100 mg increase | -0.05 (-0.13, 0.03) | 0.21 | **-0.16 (-0.04, -0.29)** | **0.02** | -0.16 (-0.08, 0.15) | 0.51 |
| Immunosuppression, per medication increase | 4.71 (-0.24, 9.7) | 0.061^+^ | - | - | - | - |
| Proportion of intermediate monocytes, 1% increase | **0.61 (0.24, 0.98)** | **0.003** | 0.60 (-0.22, 1.42) | 0.12 | 0.38 (-0.09, 0.84) | 0.11 |
| Table 1A. Logistic regression modelling showing association between proportion of intermediate monocytes and moderate-severe renal arteriosclerosis (>25%) adjusting for PREVENT 10-year ASCVD risk scores | | | | | | |
| **All Patients (n=26)** | | | **New LN (n=9)** | | **LN Flares (n=17)** | |
| PREVENT 10-year ASCVD risk estimate | 0.85 (0.48, 1.12) | 0.41 | NA* | - | 0.87 (0.48, 1.20) | 0.49 |
| Proportion of intermediate monocytes, 1% increase | **1.1 (1.02, 1.20)** | **0.03** | NA* | - | 1.06 (0.99, 1.16) | 0.12 |

**Small sample size and only one event occurred therefore, odds ratio and 95% CIs not precise. Significant p-values <0.05 shown in bold font.*
