# Supplementary material for: Intermediate monocytes in blood correlate with subclinical vascular changes in lupus nephritis
Source: Lupus Sci Med. 2025 Feb 6;12(1):e001432. doi: 10.1136/lupus-2024-001432 (PMC11804201; doi:10.1136/lupus-2024-001432)
Supplement: online supplemental file 3 [file lupus-12-1-s003.docx]

| Supplementary File 3. Sequential Linear Regression Models Showing Association between the Proportion of Intermediate (CD16+ CD14+) Monocytes & renal arteriosclerosis, n=26 | | | |
| --- | --- | --- | --- |
|  |  | Proportion of Intermediate Monocytes per 1% increase | |
| **Model**  **(Variables included in the respective models)** | | **HR (95% CIs)** | **p** |
| **Model 1**  (Proportion of Intermediate (CD16+ CD14+) Monocytes per 1% increase) | | **0.59 (0.22-0.95)** | **0.003** |
| **Model 2**  (Model 1 + PREVENT ASCVD Risk Score per 1 unit increase) | | **0.59 (0.21-0.96)** | **0.004** |
| **Model 3**  (Model 2 + NIH LN Chronicity Index Scores per 1 unit increase + NIH LN Activity Index Scores per 1 unit increase) | | **0.56 (0.16-0.95)** | **0.008** |
| **Model 4**  (Model 3 + Immunosuppression per medication increase) | | **0.61 (0.25-0.97)** | **0.002** |
| **Model 5**  (Model 4 + Prednisone per 1 mg increase + Hydroxychloroquine per 100 mg increase) | | **0.61 (0.24, 0.98)** | **0.003** |

**Small sample size and only one event occurred therefore, odds ratio and 95% CIs not precise. Significant p-values <0.05 shown in bold font.*
