# Supplementary material for: Intermediate monocytes in blood correlate with subclinical vascular changes in lupus nephritis
Source: Lupus Sci Med. 2025 Feb 6;12(1):e001432. doi: 10.1136/lupus-2024-001432 (PMC11804201; doi:10.1136/lupus-2024-001432)
Supplement: online supplemental file 4 [file lupus-12-1-s004.docx]

**
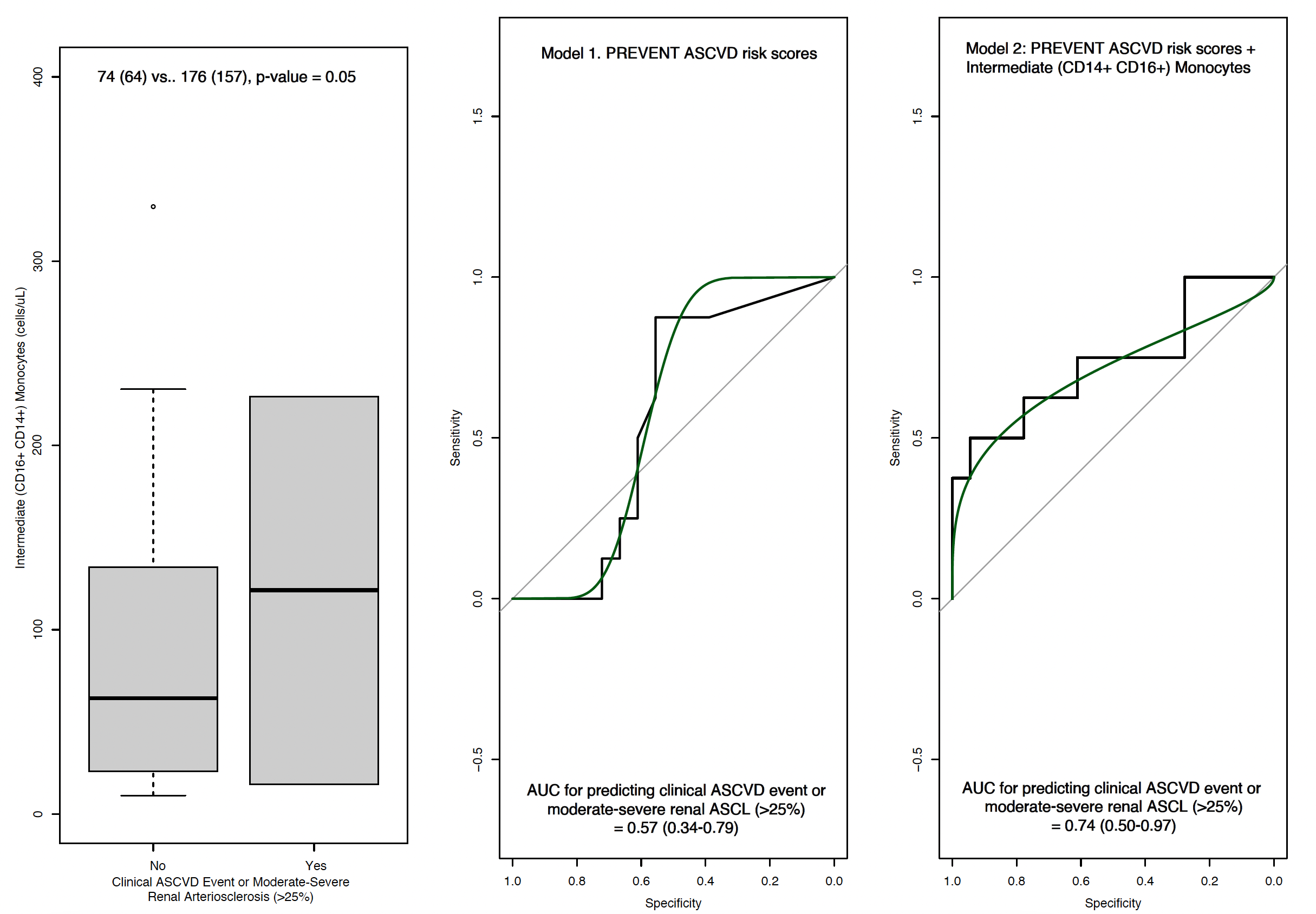
Supplementary File 4. Sensitivity analysis examining associations between intermediate (CD16+ CD14+) monocyte proportion and clinical ASCVD event or moderate-severe renal arteriosclerosis (ASCL) >25%; and area-under-the-curve (AUC) for predicting clinical ASCVD event or moderate-severe renal arteriosclerosis (ASCL) >25%**

Specificity

Specificity

Specificity

AUC for predicting ASCVD or renal ASCL >25% = 0.74 (0.50-0.97)

AUC for predicting ASCVD or renal ASCL >25% = 0.57 (0.34-0.79)

Sensitivity

Sensitivity

74 (64) vs. 176 (157), p-value = 0.05

Clinical ASCVD or renal ASCL >25%

Model 1: PREVENT ASCVD Risk Scores

Model 2: PREVENT Scores + CD16+ CD14+ Monocytes

Intermediate (CD16+ CD14+) Monocytes (cells/uL)
